# Biomechanical comparison of single- and double-bundle medial patellofemoral ligament reconstruction

**DOI:** 10.1186/s13018-017-0530-2

**Published:** 2017-02-13

**Authors:** Qing Wang, Wenhan Huang, Daozhang Cai, Huayang Huang

**Affiliations:** 1 Southern Medical University, 1838 North Guangzhou Avenue, Guangzhou, 510515 China; 2 Department of Orthopedics, Guangzhou General Hospital of Guangzhou Military Command, 111 Liu-hua Avenue, Guangzhou, 510010 China; 3 Department of Orthopaedics and Traumatology, Prince of Wales Hospital, Chinese University of Hong Kong, Hong Kong, China; 4 Department of Orthopedics, Academy of Orthopedics Guangdong Province, The Third Affiliated Hospital of Southern Medical University, 183 Zhongshan Avenue West, Guangzhou, 510665 China

**Keywords:** Medial patellofemoral ligament reconstruction, Biomechanics, Single bundle, Double bundle

## Abstract

**Background:**

Recurrent patellar dislocation is common clinically, primarily in adolescents. However, the biomechanical properties of single- and double-bundle medial patellofemoral ligament (MPFL) reconstruction remain poorly understood.

**Methods:**

Six fresh frozen adult cadaveric knee specimens were obtained for this study. Each specimen was fixed at 0° to test the force needed when the patella was laterally shifted 10 mm at a speed of 0.5 mm/s, and the test was repeated three times. This test was repeated when knee flexion was at 0°, 15°, 30°, 45°, 60°, and 90°. All six specimens were tested in four statuses, including MPFL intact, MPFL torn, single-bundle MPFL reconstruction, and double-bundle MPFL reconstruction.

**Results:**

Similar force is required in these MPFL statuses at 0° of flexion, except for the MPFL torn group with a smaller force (45.5 ± 9.6 N, *p* < 0.05). The force required in the MPFL torn group reduced from 12.8 to 38.8% compared to other groups, at 0°, 15°, 30°, and 45° of flexion angles. At the flexion of 15°, the double-bundle reconstruction group required a statistically greater force (85.9 ± 10.1 N) compared to the single-bundle reconstruction group (74.0 ± 7.9 N). Interestingly, no statistical difference was found at flexions of 60° and 90° in these four groups.

**Conclusions:**

Both single-bundle and double-bundle MPFL reconstruction can restore the stability of the patella. The double-bundle reconstruction has an angular synergy effect that simulates the MPFL wide footprint in the patella, which enables it to have greater capacity to resist patellar dislocation before the patella entering the femoral trochlea at a smaller flexion angle.

## Background

Recurrent patellar dislocation is common clinically, primarily in adolescents [1, 2]. This instability seriously affects patients’ functional movement in daily life. It is more common in patients whose medial support structure poorly heals in the knee joint after traumatic dislocation or in patients who have abnormal anatomical structures that can easily be dislocated [3].

From the anatomical and biomechanical studies of MPFL, it is found that MPFL is the primary passive restraint maintaining static stability that resists the dislocation of the patella and controls patellar tracking, providing approximately 53–60% of restraining force [2, 4]. Patients with patellar dislocation often have torn MPFL, as revealed by MRI [5]. Thus, MPFL reconstruction has great clinical implications for the treatment of patellar dislocation [6].

Conservative treatment for the recurrent patellar dislocation is less effective, and many surgical treatments have been advocated at present [7]. There have been some disputes about graft tension, tunnel location, graft fixation angles, and so on in MPFL reconstruction [8]. For example, over-tight MPFL grafts can lead to increased graft tension, limited range of motion in the knee joint, graft rupture, and iatrogenic patellar dislocation [9]. The key to successful reconstruction is to restore patellar original anatomical morphology. Since the locations of structures attaching to the medial femoral condyle vary, the relationship between MPFL near the patellar side and vastus medialis obliquus (VMO) is complex. Therefore, it causes certain controversies and confusions in MPFL anatomical origins, insertions, and the relationship between MPFL and the oblique beam tendon of VMO. These confusions will cause difficulties in MPFL reconstruction, leading to uncertain postoperative effects. The double-bundle MPFL reconstruction was developed recently to simulate anatomical structures by using two patellar tunnels [10]. However, the biomechanical properties of double-bundle MPFL reconstruction remains poorly understood. Consequently, in this study we attempted to evaluate the biomechanics of isometric single-bundle MPFL reconstruction and anatomic double-bundle MPFL reconstruction.

## Methods

**Fig. 1:**
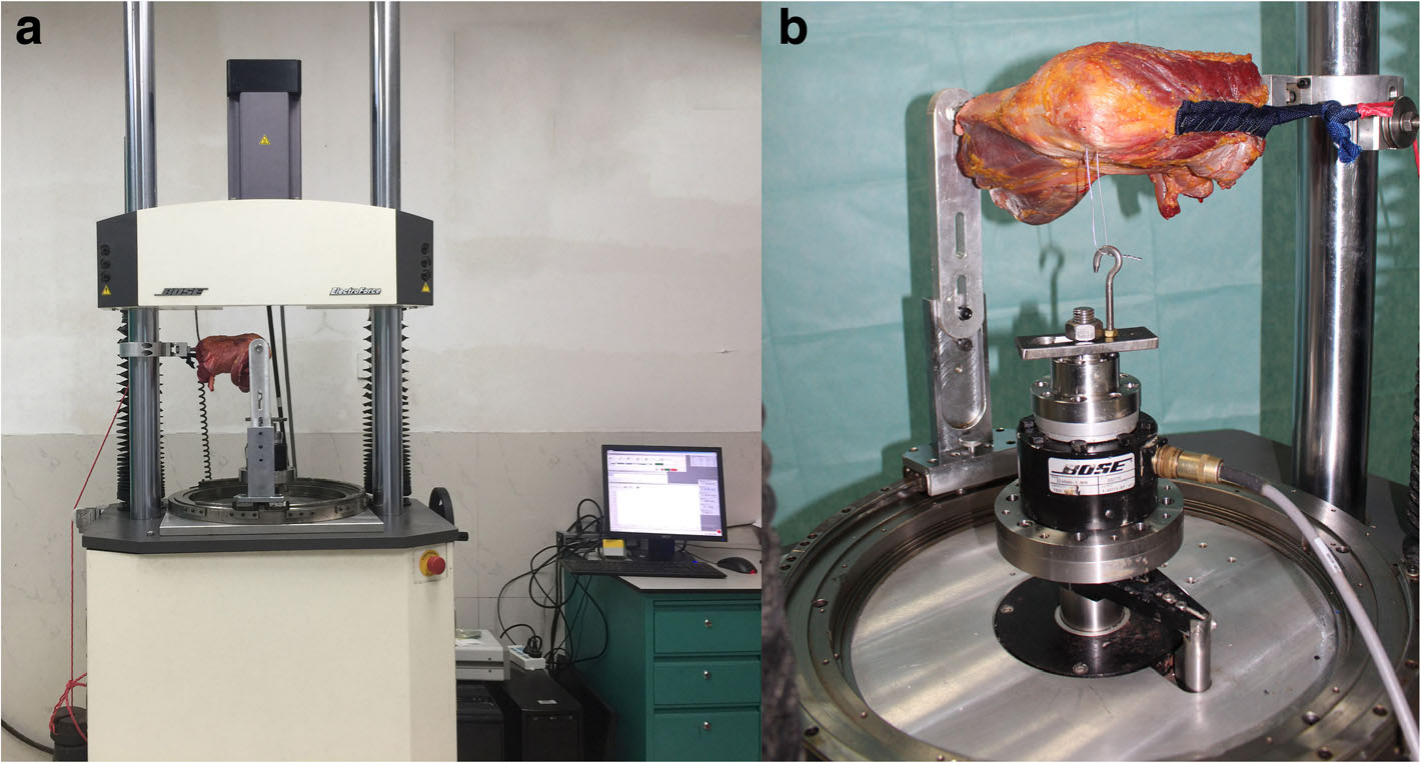
Force collected by the biomechanical material dynamic mechanical testing system. **a** Overview of the testing system. **b** The experimental setup

Six fresh frozen adult cadaveric knee specimens were obtained for this study. These specimens were comprised of 15-cm distal femurs, 15-cm proximal tibias, and their surrounding soft tissue structures. The average ages of the subjects were 57 ± 8 years old (range 48–72 years old). Cadaveric specimens had no prior surgery to the knee joint area. Deformities and apparent kinematic alterations when extended and flexed were not observed in these specimens. All operations were performed by one experienced orthopedist. A midline incision of the knee joint was performed, and the skin and subcutaneous fat were resected, retaining the ligament structures, joint capsules, and distal quadriceps. In order to remove the tibial and femoral bone marrow tissue, electric drill reaming was performed. Thus, a 9-mm diameter iron screw (the length of both the distal femur and proximal tibia was 20 cm) was inserted into the pulp cavity after drying. Then, polymethyl methacrylate (PMMA) was used for the fixation of the screw (Fig. 1).

A No. 2 Ethibond suture was used to suture the nylon string on the distal end of the distal quadriceps (simulating the contraction of the distal quadriceps through the suspension of the pulley). Two parallel holes were drilled from the lateral margin of the patella to the geometric center, and a steel wire was lead through the parallel hole. In our study, a biomechanical material dynamic mechanical testing system (Bose 520, Australia) was used for data collection. A nylon rope through the pulley was used to suspend 170 N of the axial load on the quadriceps (simulating quadriceps contraction). The steel wire through the patella was linked with the hook of the machine arm equipped with the system (providing the lateral shift of the patella).

The test protocol is based on previous studies [11, 12]. First, each specimen was fixed at 0° to test the force needed when the patella was laterally shifted 10 mm at the speed of 0.5 mm/s, and the test was repeated three times. This test was repeated when knee flexion was at 0°, 15°, 30°, 45°, 60°, and 90°. All six specimens were tested in the four states, including MPFL intact, MPFL torn, single-bundle MPFL reconstruction, and double-bundle MPFL reconstruction.

### Surgical procedure

#### MPFL torn

Through two 3-cm incisions, the medial border of the patella and distal femur slightly distal to the adductor tubercle was exposed. The MPFL was torn near these two incisions.

#### Semitendinosus tendon preparation

The semitendinosus tendon was resected through a 3-cm incision over the insertion of the pesanserinus tendons. The fascia of the sartorius muscle was pushed out through an oblique incision, and the tendon was exposed and resected. The semitendinosus tendon was used (at least 20 cm), as its greater length and volume allowed for better graft manipulation.

### Single-bundle reconstruction

#### Femoral tunnel

The starting footprint in the femur of MPFL between the femur condyle and adductor tubercle was exposed to determine the footprint center, and from medial to the lateral direction, a guild-wire was used horizontally through an anterior cruciate ligament tibial locator. After obtaining a suitable position, a hollow drill (6 mm in diameter) was used to make the femoral tunnel.

#### Patella tunnel

**Fig. 2:**
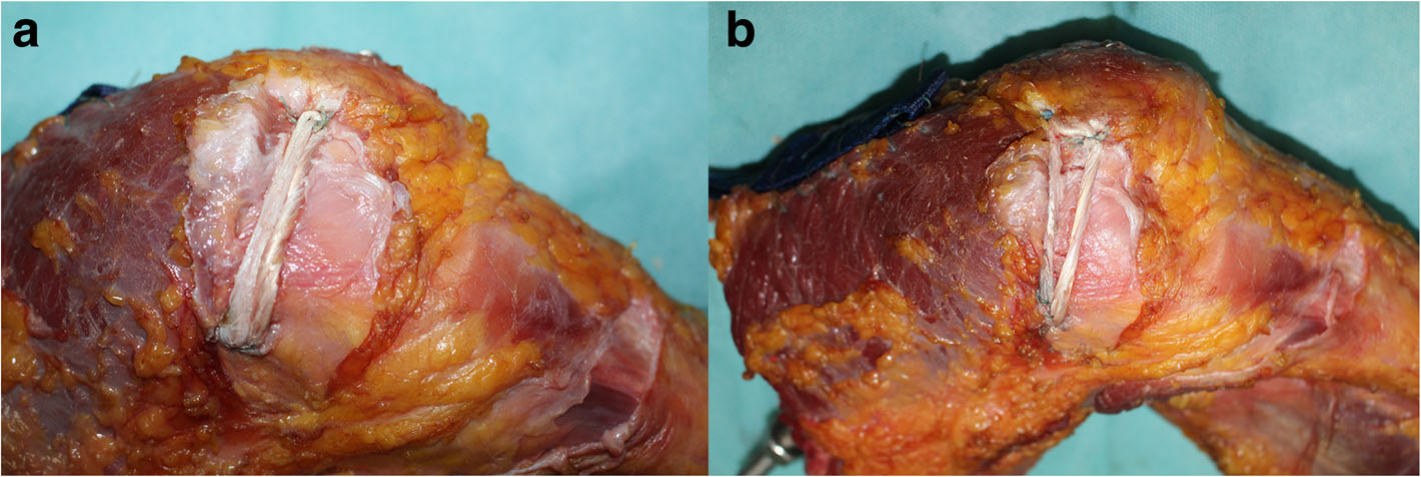
**a** MPFL reconstruction with single bundle on the *left*; **b** double-bundle MPFL reconstruction on the *right*

Half of the border of the medial patella was exposed to determine the center of the medial patella (Fig. 2a). Then, at the midpoint of the medial patella, a 2-mm diameter needle beveled at a 30° angle with the transverse axis of the patella was drilled by an anterior cruciate ligament tibia locator. After obtaining a suitable position, another needle (2 mm in diameter) was also drilled into the patella (30°) on the other side of the transverse axis of the patella. Then, two suture anchors (3.5 mm in diameter) were driven into the medial edge of the patella. These suture anchors were sutured with the semitendinosus, and the other sides of the suture anchors were instructed into the femoral tunnel by a nose needle. Finally, the suture anchors were given 2 N of tension on the traction and were fixed by an interference screw (6 mm in diameter).

### Double-bundle reconstruction

*Femoral tunnel*: the same tunnel as the single-bundle reconstruction.

*Patella tunnel*: a low straight beam was sutured by the inferior suture anchor and wire in the single bundle reconstruction (Fig. 2b). The superior-oblique beam was sutured 7 mm away from the upper pole of the patella. A guide wire (2 mm in diameter) was beveled at an angle of 60° with the vertical axis of the patella (at an angle of 30° with the transverse axis of the patella) and drilled into the patella. After confirming the proper placement of the guide wire, suture anchors (3.5 mm in diameter) were driven into the patella. Then, the prepared folded semitendinosus tendon was sutured with these two suture anchors. The other sides of the suture anchors were instructed into the femoral tunnel by a nose needle. Finally, the suture anchors were given 2 N of tension on the traction and were fixed by a 7-mm-diameter interference screw (in case of unstable fixation caused by the distensible femoral tunnel).

After fixation, the patellar position was verified through arthroscopic image and by the mobility of the patella at around one quarter of its size.

SPSS 19.0 (Chicago, USA) was used for data processing. The force required at different flexion angles in one group was analyzed using one-way ANOVA, and the level of statistical significance was set at *P* < 0.05. The force required with the same flexion angle among different groups was also analyzed using one-way ANOVA, and the level of statistical significance was also set at *P* < 0.05. When a statistical significant difference was detected in the analysis, a post hoc pair-wise comparison by Student-Newman-Keuls test was performed.

## Results

In normal knees, the force required to shift the patella 10 mm laterally at the 0° of the flexion is 74.3 ± 10.7 N (Table 1). Most of the time, this force increased as the flexion angle increased (*P* < 0.05), except for the 45° flexion angle.

**Table 1:** The force required when the patella shifts 10 mm laterally

Group	0°	15°	30°	45°	60°	90°
MPFL intact	74.3 ± 10.7^†#Δ^	74.7 ± 10.6^#Δ^	92.3 ± 12.2*	86.5 ± 10.1	96.3 ± 10.0*	101.5 ± 13.8*
MPFL cut	45.5 ± 9.6^#Δ^	48.9 ± 8.9^#Δ^	53.5 ± 9.7^#Δ^	57.3 ± 7.6^#Δ^	84.0 ± 11.5	85.5 ± 10.9
MPFL single-R	71.7 ± 8.0^†‡#Δ^	74.0 ± 7.9	97.9 ± 12.6*	90.6 ± 11.1*	89.4 ± 9.3*	97.7 ± 12.1*
MPFL double-R	74.8 ± 8.0^†#Δ^	85.9 ± 10.1^Δ^	91.5 ± 8.4^Δ^	84.7 ± 8.9^Δ^	92.1 ± 10.1^Δ^	106.3 ± 13.5^Δ^

In the same group, *Others vs. 15° *P* < 0.05; ^†^Others vs. 30° *P* < 0.05; ^‡^Others vs. 45° *P* < 0.05; ^#^Others vs. 60° *P* < 0.05; ^Δ^Others vs. 90° *P* < 0.05

With respect to the MPFL torn group, the force required to shift the patella 10 mm laterally has no statistical difference at lower flexion angles (from 0° to 45°), while at flexions of 60° and 90°, the force required sharply increased (*P* < 0.05).

In the MPFL single-bundle reconstruction group, a sharper increase in force was observed after 15° of flexion. However, after that, no significant difference was detected in higher flexion angles. In the MPFL double-bundle reconstruction group, the force required at 30°, 60°, and 90° was greater than that at 0° (*P* < 0.05). The force required at 90° (106.3 ± 13.5 N) was significantly higher than that in any of the other angles in this group.

**Fig. 3:**
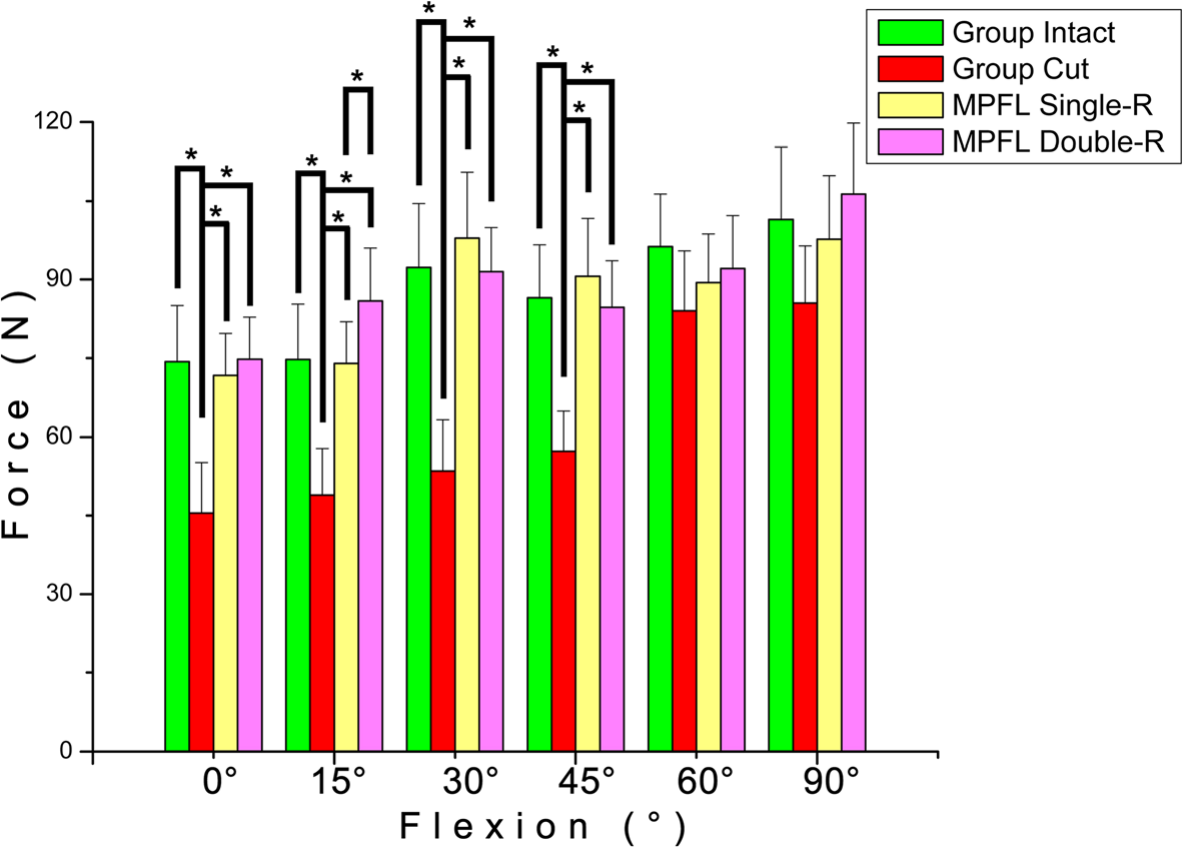
The force required when the patella shifts 10 mm laterally

When comparing the force required in different groups at the same flexion angle (Fig. 3), a similar trend was found in the first four angles. Furthermore, it was found that the MPFL torn group requires a significantly smaller force to permit a 10-mm patella lateral shift at flexions of 0°, 15°, 30°, and 45°, compared to the other groups. The force required in the MPFL torn group reduced from 12.8 to 38.8% at these four angles, compared to the other groups. At a flexion of 15°, the double-bundle reconstruction group required a greater force, compared to the single-bundle reconstruction group (*P* < 0.05). Interestingly, no statistical difference was found at flexions of 60° and 90°. During the entire flexion, no significant difference was found between the intact group and deficient groups.

## Discussion

In our study, we found that both single-bundle and double-bundle MPFL reconstruction can restore the stability of the patella. In addition, these results imply that double-bundle reconstruction greatly enhances the capacity to resist the patella’s dislocation before the patella entering the femoral trochlea. This indicates that double-bundle reconstruction has angular synergy effects, simulating the MPFL wide footprint in the patella at a smaller flexion angle [13]. Regarding the precision, in our study in an intact knee joint when knee flexion is at 0° and 15°, a 10-mm patellar shift requires 74.3 ± 10.7 N and 74.7 ± 10.6 N of lateral stress, respectively; and it requires 101.5 ± 13.8 N when knee flexion is at 90°. This is close to the results of other cadaveric patellar biomechanical models, which support the validity of the current experimental setup [2, 14, 15].

Results in current study show that both single- and double-bundle MPFL reconstruction can help restore the biomechanics of the patellofemoral joint. The forces required for 10-mm lateral shift is greater in the double-bundle MPFL reconstruction than that in the single-bundle MPFL reconstruction when knee flexion was at 15°. This is possibly due to the synergism of the bundles at that degree of knee flexion, as they form an angle of 12° to 15° in the double-bundle MPFL reconstruction. Another possible explanation would be that the extensive footprint of MPFL is imitated in the double-bundle reconstruction, which requires larger resistance to the patellar shift before the patella gliding into the femoral trochlea.

Previous study has shown that the patella’s resistance to the outer edge stress is at the lowest level during knee flexion at 20° to 30° [16]. In fact, from the literature such angles are clinically common angles for patellar dislocation [17–19]. Based on these biomechanical results, we can speculate that double-bundle MPFL reconstruction has advantages in preventing patellar lateral recurrent dislocation after operation. In some clinical studies, it was shown that the double-bundle reconstruction achieved far better clinical outcomes in the long-term follow-up compared to single-bundle reconstruction [20].

No statistical differences were found in force required among groups at flexion of 60° and 90°. It is reasonable since the medial facet of lateral femur condyle resisted the lateral shift after entering femoral trochlea at higher flexion angle, despite the torn MPFL. Meanwhile, the force required was increasing in all groups because of the resistance from femur condyle.

Kang et al. researched the anatomy of MPFL and put forward the concept of bi-functional bundles [10]. It has been reported that double-bundle anatomical reconstruction is consistent with the patellofemoral ligament in terms of anatomical characteristics [21]. The application of the double-bundle anatomical reconstruction and reconstruction of the straight bundle below the patellofemoral ligament can help restore a low level of stability. In the case of knee flexion that occurs earlier before the patella gliding into the femoral trochlea, the contraction of the upper tilt bundle and medial femoral tilt muscle bundle can stabilize the patella [13]. The coupling of the upper tilt bundle reconstruction and medial femoral oblique muscle can strengthen the dynamic and static stability of the knee joint.

The limitations of this study include the small number of samples, samples from older subjects who had different geometric structures, biomechanical properties in the patella compared to those from youth. This may have effects on the experiment. Even though no pathological characteristics or abnormal patella rotation were observed in the included samples, VMO and femoral trochlear dysplasia may still disrupt the imitation of patellar dislocation.

## Conclusions

We found that both single-bundle and double-bundle MPFL reconstruction can restore the stability of patella and that the force required to shift the patella 10 mm laterally in the double-bundle MPFL reconstruction is higher than that in the single-bundle reconstruction at 15°. Furthermore, double-bundle reconstruction has angular synergy effects that simulate the MPFL wide footprint in the patella. This enables it to have greater capacity to resist patellar lateral dislocation before the patella entering the femoral trochlea at a smaller flexion angle. Further studies should be done to verify its clinical efficacy.

## Abbreviations

MPFL: Medial patellofemoral ligament
PMMA: Polymethyl methacrylate
VMO: Vastus medialis oblique

## References

[1] Nomura E, Inoue M, Osada N (2005). Anatomical analysis of the medial patellofemoral ligament of the knee, especially the femoral attachment. Knee Surg Sports Traumatol Arthrosc.

[2] Amis A, Firer P, Mountney J, Senavongse W, Thomas N (2003). Anatomy and biomechanics of the medial patellofemoral ligament. Knee.

[3] Nomura E, Horiuchi Y, Kihara M (2000). A mid-term follow-up of medial patellofemoral ligament reconstruction using an artificial ligament for recurrent patellar dislocation. Knee.

[4] Mikashima Y, Kimura M, Kobayashi Y, Miyawaki M, Tomatsu T (2006). Clinical results of isolated reconstruction of the medial patellofemoral ligament for recurrent dislocation and subluxation of the patella. Acta Orthopaedica Belgica.

[5] Smirk C, Morris H (2003). The anatomy and reconstruction of the medial patellofemoral ligament. Knee.

[6] Nelitz M, Williams SRM (2014). Anatomic reconstruction of the medial patellofemoral ligament in children and adolescents using a pedicled quadriceps tendon graft. Arthrosc Tech.

[7] Ji G, Wang S, Wang X, Liu J, Niu J, Wang F. Surgical versus nonsurgical treatments of acute primary patellar dislocation with special emphasis on the MPFL injury patterns. J Knee Surg. 2016;14. Epub 2016/09/15. 10.1055/s-0036-1592151 27626368

[8] Burrus MT, Werner BC, Cancienne JM, Gwathmey FW, Diduch DR. MPFL graft fixation in low degrees of knee flexion minimizes errors made in the femoral location. Knee Surgery, Sports Traumatology, Arthroscopy. 2016:1-7. In press. doi: 10.1007/s00167-016-4111-4. 10.1007/s00167-016-4111-4 27085363

[9] Thaunat M, Erasmus PJ (2009). Management of overtight medial patellofemoral ligament reconstruction. Knee Surg, Sports Traumatol, Arthrosc..

[10] Kang HJ, Wang F, Chen BC, Su YL, Zhang ZC, Yan CB (2010). Functional bundles of the medial patellofemoral ligament. Knee Surg, Sports Traumatol, Arthrosc.

[11] Bedi H, Marzo J (2010). The biomechanics of medial patellofemoral ligament repair followed by lateral retinacular release. Am J Sports Med.

[12] Sadigursky D, Gobbi RG, Pereira CA, Pecora JR, Camanho GL (2012). Biomechanical access method for analyzing isometricity in reconstructing the medial patellofemoral ligament. Rev Bras Ortop.

[13] Ji G, Wang F, Zhang Y, Chen B, Ma L, Dong J (2012). Medial patella retinaculum plasty for treatment of habitual patellar dislocation in adolescents. Int Orthop.

[14] Christoforakis J, Bull AM, Strachan RK, Shymkiw R, Senavongse W, Amis AA (2006). Effects of lateral retinacular release on the lateral stability of the patella. Knee Surgery, sports Traumatol, Arthroscopy.

[15] Mountney J, Senavongse W, Amis AA, Thomas NP (2005). Tensile strength of the medial patellofemoral ligament before and after repair or reconstruction. J Bone Joint Surg.

[16] Senavongse W, Amis AA (2005). The effects of articular, retinacular, or muscular deficiencies on patellofemoral joint stability: a biomechanical study in vitro. J Bone Joint Surg.

[17] Colvin AC, West RV (2008). Patellar instability. J Bone Joint Surg Am.

[18] Draper CE, Besier TF, Fredericson M, Santos JM, Beaupre GS, Delp SL (2011). Differences in patellofemoral kinematics between weight-bearing and non-weight-bearing conditions in patients with patellofemoral pain. J Orthop Res.

[19] Powers CM, Ward SR, Fredericson M, Guillet M, Shellock FG (2003). Patellofemoral kinematics during weight-bearing and non-weight-bearing knee extension in persons with lateral subluxation of the patella: a preliminary study. J Orthop Sports Phys Ther.

[20] Wang CH, Ma LF, Zhou JW, Ji G, Wang HY, Wang F (2013). Double-bundle anatomical versus single-bundle isometric medial patellofemoral ligament reconstruction for patellar dislocation. Int Orthop.

[21] Panagiotopoulos E, Strzelczyk P, Herrmann M, Scuderi G (2006). Cadaveric study on static medial patellar stabilizers: the dynamizing role of the vastus medialis obliquus on medial patellofemoral ligament. Knee Surg, Sports Traumatol, Arthrosc.

